# Clinical characterization of common pathogenic variants of *SOD1*-ALS in Germany

**DOI:** 10.1007/s00415-024-12564-1

**Published:** 2024-08-14

**Authors:** Maximilian Wiesenfarth, Yalda Forouhideh-Wiesenfarth, Zeynep Elmas, Özlem Parlak, Ulrike Weiland, Christine Herrmann, Joachim Schuster, Axel Freischmidt, Kathrin Müller, Reiner Siebert, Kornelia Günther, Elke Fröhlich, Antje Knehr, Tatiana Simak, Franziska Bachhuber, Martin Regensburger, Susanne Petri, Thomas Klopstock, Peter Reilich, Florian Schöberl, Peggy Schumann, Peter Körtvélyessy, Thomas Meyer, Wolfgang P. Ruf, Simon Witzel, Hayrettin Tumani, David Brenner, Johannes Dorst, Albert C. Ludolph

**Affiliations:** 1 Department of Neurology, Ulm University, Oberer Eselsberg 45, 89081 Ulm, Germany; 2 German Centre for Neurodegenerative Diseases (DZNE) Site Ulm, 89081 Ulm, Germany; 3 Institute of Human Genetics, Ulm University and Ulm University Medical Center, 89081 Ulm, Germany; 4 Department of Molecular Neurology, Friedrich-Alexander-Universität Erlangen-Nürnberg (FAU), 91054 Erlangen, Germany; 5 Deutsches Zentrum Immuntherapie (DZI), University Hospital Erlangen, 91054 Erlangen, Germany; 6 Department of Neurology, Hannover Medical School, 30625 Hannover, Germany; 7 Department of Neurology with Friedrich-Baur-Institute, LMU University Hospital, LMU Munich, 80336 Munich, Germany; 8 German Centre for Neurodegenerative Diseases (DZNE) Site Munich, 81377 Munich, Germany; 9 Munich Cluster for Systems Neurology (SyNergy), 81377 Munich, Germany; 10 Ambulanzpartner Soziotechnologie GmbH, 13353 Berlin, Germany; 11 Department of Neurology, Center for ALS and other Motor Neuron Disorders, Charité—Universitätsmedizin Berlin, Corporate Member of Freie Universität Berlin, Humboldt-Universität zu Berlin, Berlin Institute of Health, 13353 Berlin, Germany; 12 German Centre for Neurodegenerative Diseases (DZNE) Site Magdeburg, 39120 Magdeburg, Germany

**Keywords:** Amyotrophic lateral sclerosis, *SOD1*, Motor neuron disease, Clinical phenotype, Tofersen

## Abstract

Pathogenic variants in the Cu/Zn superoxide dismutase (*SOD1*) gene can be detected in approximately 2% of sporadic and 11% of familial amyotrophic lateral sclerosis (ALS) patients in Europe. We analyzed the clinical phenotypes of 83 *SOD1*-ALS patients focusing on patients carrying the most frequent (likely) pathogenic variants (R116G, D91A, L145F) in Germany. Moreover, we describe the effect of tofersen treatment on ten patients carrying these variants. R116G patients showed the most aggressive course of disease with a median survival of 22.0 months compared to 198.0 months in D91A and 87.0 months in L145F patients (HR 7.71, 95% CI 2.89–20.58 vs. D91A; p < 0.001 and HR 4.25, 95% CI 1.55–11.67 vs. L145F; p = 0.02). Moreover, R116G patients had the fastest median ALSFRS-R progression rate with 0.12 (IQR 0.07–0.20) points lost per month. Median diagnostic delay was 10.0 months (IQR 5.5–11.5) and therefore shorter compared to 57.5 months (IQR 14.0–83.0) in D91A (p < 0.001) and 21.5 months (IQR 5.8–38.8) in L145F (p = 0.21) carriers. As opposed to D91A carriers (50.0%), 96.2% of R116G (p < 0.001) and 100.0% of L145F (p = 0.04) patients reported a positive family history. During tofersen treatment, all patients showed a reduction of neurofilament light chain (NfL) serum levels, independent of the *SOD1* variant. Patients with *SOD1*-ALS carrying R116G, D91A, or L145F variants show commonalities, but also differences in their clinical phenotype, including a faster progression rate with shorter survival in R116G, and a comparatively benign disease course in D91A carriers.

**Supplementary Information:**

The online version contains supplementary material available at 10.1007/s00415-024-12564-1.

## Introduction

A pathogenic variant in the Cu/Zn superoxide dismutase (*SOD1*) gene was first described in 1993 [1] as a cause of familial Amyotrophic lateral sclerosis (ALS), a neurodegenerative disease characterized by progressive muscle weakness and a severely reduced life expectancy of about 2–5 years after onset by irreversible affection of the upper and lower motor neurons [2]. Overall, a positive family history is reported in about 5–10% of ALS patients [3, 4]. Pathogenic variants of the *SOD1* gene are the most common mutations in Asia and, after *C9orf72* hexanucleotide repeat expansions, in Europe [5–7], occurring in approximately 2% of sporadic [8] and 11% of familial cases [9]. There are more than 230 different ALS-causing variants in the *SOD1* gene with heterogeneous clinical phenotypes and large differences with regard to prevalence across regions [10]. For example, in the United States, A5V (exon 1) has been described to be the most common pathogenic variant of *SOD1*-ALS, accounting for approximately 50% of all *SOD1* cases, and has been thoroughly characterized by Cudkowicz et al. [11–13], whereas in China, H47R (exon 2) is the most frequent pathogenic variant [14]. In Germany, pathogenic variants of *SOD1-*ALS are mainly localized in exons 4 and 5, with R116G (exon 4) representing the most frequent ALS-associated mutation, followed by L145F (exon 5) and D91A (exon 4) [14, 15]. As a common pathomechanism of *SOD1*-associated ALS, a toxic gain of function of mutant SOD1 protein has been proposed [10, 16, 17]. Of note, in addition to pharmacological treatment with riluzole [18], edaravone [19], and sodium phenylbutyrate/taurursodiol [20], the antisense oligonucleotide (ASO) tofersen, which reduces the synthesis of SOD1 protein by RNase H-dependent degradation of *SOD1* messenger RNA [21], has been approved by the U.S. Food and Drug Administration (FDA) for patients with *SOD1*-ALS in April 2023. In Europe, tofersen has been recently approved by the European Medicines Agency (EMA) in May 2024, but was already available via Early Access Programs (EAPs) in several countries since March 2022. Patients who participated in the EAP in Germany showed a reduction of neurofilament levels (NfL in serum and pNfH in CSF) and also promising effects on clinical progression parameters such as the ALS functional rating scale revised (ALSFRS-R) [22, 23]. However, since the effect of tofersen might depend on the *SOD1* variant, a precise characterization of different genotypes and their associated clinical phenotypes and disease courses is necessary to better estimate treatment effects. Therefore, in this study, we characterized and compared the clinical features (ALSFRS-R, progression rate, site of onset (spinal/bulbar), affection of upper and lower motor neurons (UMN or LMN), body mass index (BMI), sex, age of onset, diagnostic delay, and family history) of the most frequently occurring pathogenic variants of *SOD1*-ALS in Germany. Moreover, we present our first experiences with tofersen treatment in these patients.

## Materials and methods

### Participants

We retrospectively analyzed 83 patients, who were diagnosed with definite, probable, or possible ALS according to revised El Escorial criteria [24] between 2003 and 2019 and had a (likely) pathogenic variant of the *SOD1* gene according to the American College of Medical Genetics and Genomics and the Association for Molecular Pathology (ACMG-AMP) [25, 26]. One patient was included with a variant of uncertain significance (VUS) as this finding was classified as causative for the diseases by the treating physicians. In addition, 10 patients with *SOD1*-ALS, who received tofersen treatment in the German EAP between March 2022 and April 2023, and were carrying the most frequent (likely) pathogenic variants in the German population (R116G, D91A and L145F), were included from five specialized centers (University of Ulm, Charité Berlin, University Hospital Erlangen, Hannover Medical School, Ludwig Maximilians University Munich) of the MND-NET, a clinical and scientific network of 26 German motoneuron disease centers. The cohort contains data of patients, which have been published previously by Wiesenfarth and colleagues [22]. The cut-off for data inclusion in the cohort with 83 retrospectively analyzed patients was 2019.

The study was approved by the institutional Ethics Committee of Ulm University (application number 19/12).

### Demographic and clinical data

Demographic data included sex, age of onset, diagnostic delay (time between first paresis and diagnosis), and family history of ALS. Clinical features included site of onset (spinal/bulbar), predominant affectation of UMN or LMN, BMI at onset, ALSFRS-R [27], progression rate (points of ALSFRS-R lost per month between disease onset and last visit), and survival. Collection of demographic and clinical data was performed by physicians at specialized centers with dedicated experience in the diagnosis of ALS.

Moreover, we descriptively analyzed clinical outcome parameters, including ALSFRS-R and NfL serum levels, of 10 *SOD1*-ALS patients, who were treated in the German EAP and of whom 4 patients (R116G_1, R116G_2, R116G_3 and R116G_4) carried a R116G, 3 patients (D91A_1, D91A_2 and D91A_3) a D91A and 3 patients (L145F_1, L145F_2 and L145F_3) a L145F mutation. In D91A_1 and D91A_3 the variant was present in homozygosity whereas D91A_2 carried the variant in heterozygous state.

### DNA sequencing and analysis

DNA was extracted from blood samples and analyzed by Sanger sequencing for all coding exons and flanking 50bps of *SOD1*. Sequence analysis has been previously published [28].

### Statistical analysis

For descriptive statistics, median (IQR) or mean (SD) are given. For group comparisons, the Chi-square test was applied for nominal variables and the unpaired student’s* t*-test for continuous variables. The Mann–Whitney *U* test was used for non-normally distributed variables. Kaplan–Meier curves and log-rank test were applied to determine the effect of demographic or clinical parameters on survival. A *P*-value of ≤ 0.05 was regarded as statistically significant. As our study was explorative and hypothesis-generating, we did not perform adjustment for multiple testing. Due to the small number of cases, data of patients receiving tofersen treatment were analyzed descriptively and, therefore, no statistical tests were performed.

Statistical analyses were performed using GraphPad Prism version 10.0.2 for Windows (GraphPad Software, San Diego, California USA).

### Data availability

Individual participant data, which underlie the results reported in this article, after de-identification (text, tables, and figures) as well as the study protocol, will be available. Data will be available beginning 3 months and ending 5 years following article publication. Data will be shared with researchers who provide a methodologically sound proposal. Data will be shared for analyses to achieve the aims in the approved proposal. Proposals should be directed to maximilian.wiesenfarth@rku.de; to gain access, data requestors will need to sign a data access agreement.

## Results

### Demographic and clinical data at baseline

Overall, 83 patients with ALS carrying a variant in the *SOD1* gene were included in the study. Out of these patients, we identified the three most frequently occurring *SOD1* variants, which account for approximately half the of *SOD1*-ALS cases (50.6%) in Germany (Table 1). Accordingly, the three comparator groups consisted of 26 patients with R116G (p.Arg116Gly), 10 patients with D91A (p.Asp91Ala), of whom 6 patients carried the variant homozygously and 4 patients heterozygously, and 6 patients with L145F (p.Leu145Phe) mutations. Frequencies of *SOD1* variants in the German population and their classification according to the ACMG-AMP criteria are shown in Table 1. The clinical and demographic data of the 41 patients who were carrying variants of the *SOD1* gene other than R116G, D91A and L145F are additionally presented in Table 2.

**Table 1:** Frequencies of *SOD1* variants in the German population

*SOD1* variant NP_000445.1 (NM_000454.5)	number of patients (*n*)	frequency (%)	ACMG-AMP class
p.Arg116Gly (c.346C > G)	26	31.33	Likely pathogenic
p.Asp91Ala (c.272A > C)	10	12.05	Pathogenic
p.Leu145Phe (c.435G > C)	6	7.23	Pathogenic
p.Glu101Lys (c.301G > A)	5	6.02	Pathogenic
p.Gly73Ser (c.217G > A)	3	3.61	Likely pathogenic
p.His44Arg (c.131A > G)	2	2.41	Likely pathogenic
p.His47Arg (c.140A > G)	2	2.41	Pathogenic
p.His49Arg (c.146A > G)	2	2.41	Likely pathogenic
p.Val88Ala (c.263T > C)	2	2.41	Pathogenic
p.Ile105Phe (c.313A > T)	2	2.41	Likely pathogenic
p.Ile113Thr (c.338T > C)	2	2.41	Pathogenic
p.Val149Gly (c.446T > G)	2	2.41	Likely pathogenic
p.Val6Leu (c.16G > T)	1	1.20	Likely pathogenic
p.Leu39Val (c.115C > G)	1	1.20	Likely pathogenic
p.Glu41Gly (c.122A > G)	1	1.20	Likely pathogenic
p.Asn66Ser (c.197A > G)	1	1.20	Likely pathogenic
p.His72Tyr (c.214 > T)	1	1.20	Likely pathogenic
p.Leu85Val (c.253T > G)	1	1.20	Pathogenic
p.Leu85Phe (c.255G > C/T)	1	1.20	Pathogenic
p.Gly86Arg (c.256G > C)	1	1.20	Pathogenic
p.Asn87Lys (c.261T > A)	1	1.20	Pathogenic
p.Asn87Ser (c.260A > G)	1	1.20	Pathogenic
p.Asp102Asn (c.304G > A)	1	1.20	Pathogenic
p.Gly109Val (c.326G > T)	1	1.20	Pathogenic
p.Asp110Tyr (c.328G > T)	1	1.20	VUS
p.Ile114Thr (c.341T > C)	1	1.20	Likely pathogenic
p.Leu118Val (c.352C > G)	1	1.20	Likely pathogenic
p.Glu134Lys (c.400G > A)	1	1.20	Likely pathogenic
p.Gly148Asp (c.443G > A)	1	1.20	Pathogenic
p.Val149Ala (c.446T > C)	1	1.20	Likely pathogenic
p.Ile152Thr (c.455T > C)	1	1.20	Likely pathogenic

*ACMG-AMP* American College of Medical Genetics and Genomics and the Association for Molecular Pathology, *VUS* variant of uncertain significance

**Table 2:** Clinical features of patients with different variants of the *SOD1* gene

	R116G (*n* = 26)	D91A (*n* = 10)	L145F (*n* = 6)	Others (*n* = 41)
**Age of onset** (median, IQR)	52.0 (47.0–60.3) (*n* = 22)	50.0 (41.0–64.3) (*n* = 10)	54.0 (44.8–56.3) (*n* = 6)	48.0 (33.5–58.0) (*n* = 40)
**Sex**				
Male	69.2% (*n* = 18)	40.0% (*n* = 4)	66.7% (*n* = 4)	50.0% (*n* = 20)
Female	30.8% (*n* = 8)	60.0% (*n* = 6)	33.3% (*n* = 2)	50.0% (*n* = 20)
**Onset**				
Spinal	100.0% (*n* = 18)	100.0% (*n* = 6)	100.0% (*n* = 6)	82.1% (*n* = 32)
Bulbar	0.0% (*n* = 0)	0.0% (*n* = 0)	0.0% (*n* = 0)	17.9% (*n* = 7)
**Family history**				
Positive	96.2% (*n* = 25)	50.0% (*n* = 5)	100% (*n* = 6)	82.1% (*n* = 32)
Negative	3.8% (*n* = 1)	50.0% (*n* = 5)	0% (*n* = 0)	17.9% (*n* = 7)
**ALSFRS-R**^**a**^ (1st visit) (median, IQR)	42.5 (39.0–45.0) (*n* = 14)	37.5 (32.0–40.8) (*n* = 8)	43.0 (23.0–45.3) (*n* = 6)	38.0 (30.0–44.0) (*n* = 31)
**Progression rate** (median, IQR) (onset to last visit)	0.12 (0.07–0.20) (*n* = 14)	0.03 (0.02–0.08) (*n* = 8)	0.06 (0.04–0.14) (*n* = 6)	0.50 (0.13–1.38) (*n* = 32)
**BMI**^**b**^** in kg/m**^**2**^ (median, IQR)	26.8 (24.9–27.7) (*n* = 9)	25.2 (19.8–27.4) (*n* = 6)	26.3 (24.4–31.1) (*n* = 4)	25.8 (21.9–30.5) (*n* = 17)
**Diagnostic delay in months** (median, IQR)	10.0 (5.5–11.5) (*n* = 13)	57.5 (14.0–83.0) (*n* = 6)	21.5 (5.8–38.8) (*n* = 4)	12.0 (4.8–39.3) (*n* = 23)
**Survival in months** (median)	22.0 (*n* = 22)	198.0 (*n* = 10)	87.0 (*n* = 6)	248.0 (*n* = 38)
versus R116G (HR, 95% CI)		(0.13, 0.05–0.35)	(0.24, 0.09–0.65)	(0.43, 0.20–0.96)
versus D91A (HR, 95% CI)	(7.71, 2.89–20.58)		(9.10, 0.34–239.5)	(2.19, 0.73–6.62)
versus L145F (HR, 95% CI)	(4.25, 1.55–11.67)	(0.11, 0.00–2.92)		(1.14, 0.29–4.57)
versus others (HR, 95% CI)	(2.30, 1.04–5.12)	(0.46, 0.15–1.38)	(0.88, 0.22–3.50)	
**Predominance of UMN or LMN**				
upper motor neuron (UMN)	0.0% (*n* = 0)	20.0% (*n* = 1)	0.0% (*n* = 0)	6.3% (*n* = 1)
lower motor neuron (LMN)	14.3% (*n* = 1)	20.0% (*n* = 1)	66.6% (*n* = 2)	62.5% (*n* = 10)
classical ALS (UMN and LMN)	85.7% (*n* = 6)	60.0% (*n* = 3)	33.3% (*n* = 1)	31.3% (*n* = 5)

^a^*ALSFRS-R* ALS Functional Rating Scale-Revised

^b^*BMI* body mass index

### Variant-dependent comparison of clinical and demographic data

The clinical phenotype and demographic data of patients carrying R116G (31.3%), D91A (12.1%), L145F (7.2%) and other (likely) pathogenic variants in the *SOD1* gene are depicted in Table 2.

### Sex, age of onset, and family history

**Fig. 1:**
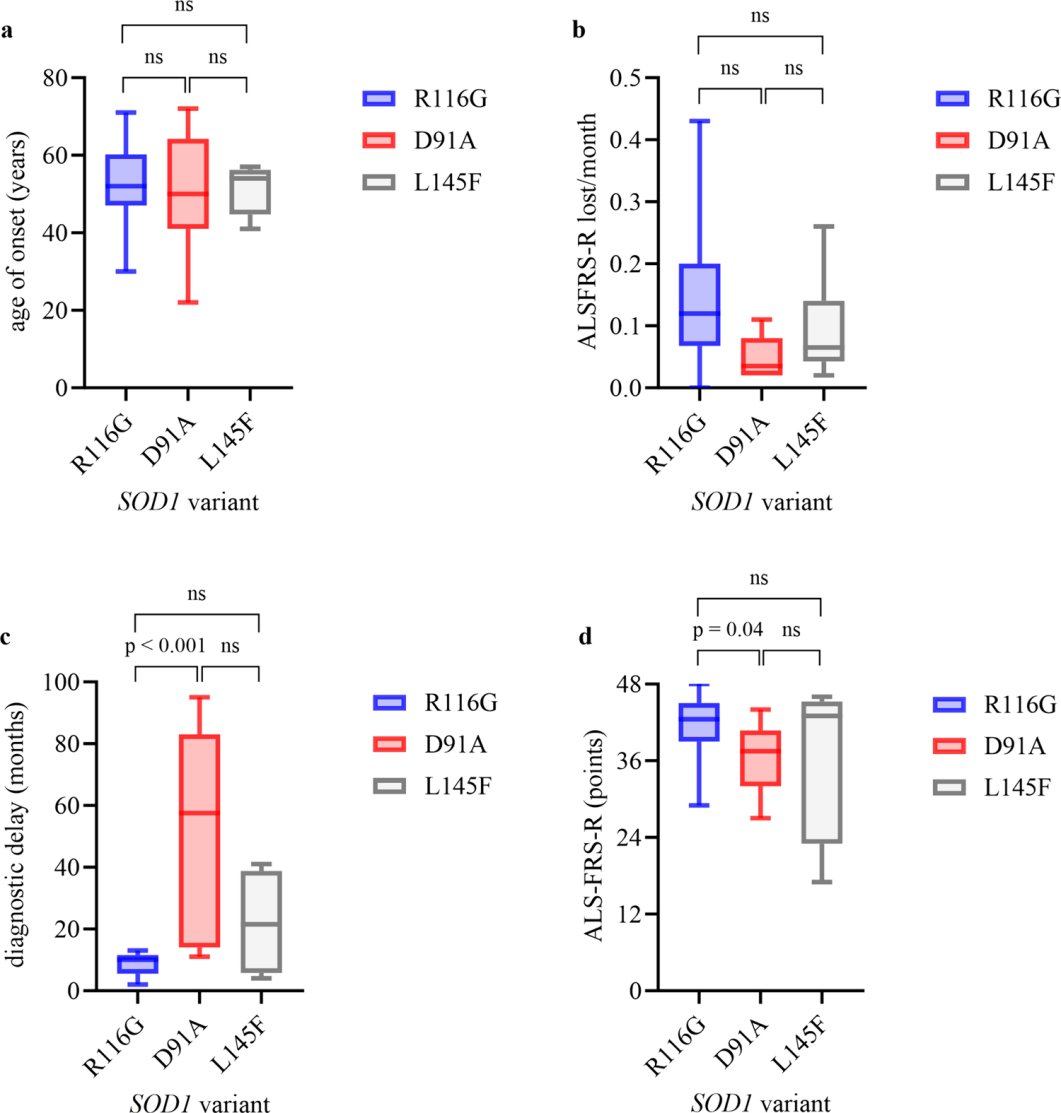
Clinical characteristics in R116G carriers vs. D91A carriers (homozygous and heterozygous) vs. L145F carriers. Boxplots show median (IQR; minimum–maximum). (**a**) age of onset (**b**) progression rate (**c**) diagnostic delay (**d**) ALSFRS-R at first visit. Experimental units *n* = number (**a**) R116G *n* = 22, D91A* n* = 10, p = 0.6814, R116G *n* = 22, L145F* n* = 6, p = 0.9239, D91A* n* = 10, L144F* n* = 6, p = 0.9798 (**b**) R116G* n* = 14, D91A* n* = 8, p = 0.0183, R116G* n* = 14, L145F* n* = 6, p = 0.2125, D91A* n* = 8, L145F* n* = 6, p = 0.3526 (**c**) R116G* n* = 13, D91A *n* = 6, p = 0.0008, R116G* n* = 13, L145F *n* = 4, p = 0.2723, D91A *n* = 6, L145F *n* = 4, p = 0.1286 (**d**) R116G* n* = 14, D91A* n* = 8, p = 0.0439, R116G* n* = 14, L145F* n* = 6, p = 0.08256, D91A* n* = 8, L145F* n* = 6, p = 0.4336. Mann–Whitney U test was used for two group comparison. A *P*-value of ≤ 0.05 was regarded as statistically significant. *ALSFRS-R* Amyotrophic lateral sclerosis functional rating scale revised

Median age of onset was 52.0 years (IQR 47.0–60.3; *n* = 22) in patients with R116G and therefore not statistically different compared to 50.0 years (IQR 41.0–64.3; *n* = 10; p = 0.68; Fig. 1a) in D91A and 54.0 years (IQR 44.8–56.3; *n* = 6) in L145F carriers (p = 0.92; Fig. 1a). Whereas in *SOD1*-ALS with R116G and L145F variants more male than female patients were affected (R116G: 69.2% vs. 30.8%; L145F: 66.7% vs. 33.3%), the D91A variant was more frequently found in females (60.0% vs. 40.0%), although these differences did not reach statistical significance. As opposed to D91A carriers (50.0%), the vast majority of R116G patients (96.2%) and all patients with L145F (100.0%) mutation reported a positive family history of ALS (D91A vs. R116G, p < 0.001; D91A vs. L145F; p = 0.04).

### Clinical phenotype

In all three variants, patients showed a spinal onset of the disease. The majority of patients carrying a R116G variant (85.7%) and D91A variant (60.0%) showed a classical phenotype with simultaneous affection of UMN and LMN, whereas a predominance of the lower motor neuron was found in 66.6% of patients with L145F variants. We did not observe any statistically significant differences with regard to median BMI between the three compared groups, although median BMI in D91A patients (25.2 kg/m^2^, IQR 19.8–27.4; *n* = 6) was slightly lower than in R116G (26.8 kg/m^2^, IQR 24.9–27.7; *n* = 9; p = 0.18) and L145F patients (26.3 kg/m^2^, IQR 24.4–31.1; *n* = 4; p = 0.26).

### ALSFRS-R, progression rate and diagnostic delay

Consistent with the survival data*, SOD1*-ALS patients with R116G variants showed a median disease progression rate of 0.12 ALSFRS-R points lost per month (IQR 0.07-0.20) between onset and last visit, which was more pronounced compared to patients with pathogenic D91A (0.03, IQR 0.02–0.08; *n* = 8; p = 0.02) and L145F variants (median 0.06, IQR 0.04–0.14; *n* = 6; p = 0.21), while D91A and L145F patients showed a quite similar, slow median progression rate (p = 0.35; Fig. 1b). Moreover, these differences in ALSFRS-R progression rate were already detected in an early phase of the disease, as a decline of 0.62 points (IQR 0.25–0.76; *n* = 14) per month in ALSFRS-R was found in patients with R116G variants compared to 0.16 (IQR 0.09–0.44; *n* = 8; p = 0.04) in patients with D91A and 0.38 with L145F variants (IQR 0.14–1.05; *n* = 6; p = 0.73) between the onset of the disease and the first visit at a MND reference center. In line with this faster disease progression, also the median delay of ALS diagnosis in patients carrying R116G variants was shorter (median 10.0 months, IQR 5.5–11.5; *n* = 13) than in patients with D91A (median 57.5 months, IQR 14.0–83.0; *n* = 6; p < 0.001) and in patients with L145F variants (median 21.5 months, IQR 5.8–38.8; *n* = 4; p = 0.27; Fig. 1c). Of note, median ALSFRS-R progression rates in patients with R116G, D91A and L145F were lower compared to patients carrying less frequent *SOD1* variants (0.50, IQR 0.13–1.38; *n* = 32; Table 2). Median ALSFRS-R at first visit was 42.5 (IQR 39.0–45.0; *n* = 14) in patients with R116G and therefore higher compared to 37.5 (IQR 32.0–40.8; *n* = 8) in D91A (p = 0.04; Fig. 1d) and similar to L145F carriers (43.0, IQR 23.0–45.3; *n* = 6; p = 0.83; Fig. 1d).

### Survival

**Fig. 2:**
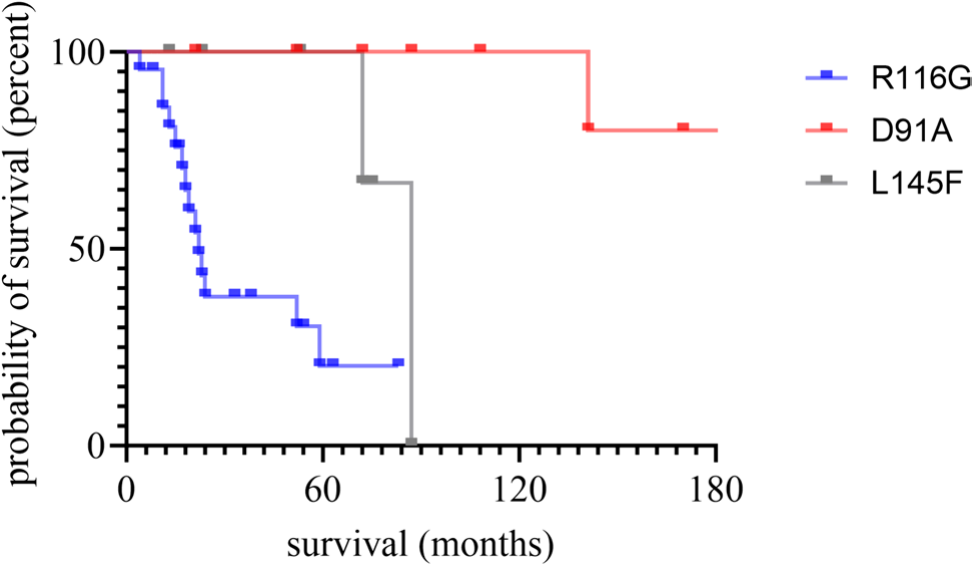
Kaplan Meier Curves for survival in R116G carriers vs. D91A carriers (homozygous and heterozygous) vs. L145F carriers. Experimental units *n* = number, R116G *n* = 22*,* D91A *n* = 10, p = 0.0002, R116G* n* = 22, L145F *n* = 6, p = 0.02. Kaplan–Meier curves and Log-rank test were applied to determine the effect of demographic or clinical parameters on survival. A *P*-value of ≤ 0.05 was regarded as statistically significant

Patients with R116G (*n* = 22) turned out to have the most aggressive form of the disease with a median survival of 22.0 months (HR 7.71, 95% CI 2.89–20.58 vs. D91A; p < 0.001 and HR 4.25, 95% CI 1.55–11.67 vs. L145F; p = 0.02; Fig. 2). Patients with D91A (*n* = 10) and L145F (*n* = 6) both had comparatively benign courses of disease with median survival of 198.0 vs. 87.0 months (D91A vs. L145F HR 0.11, 95% CI 0.00–2.92; p = 0.004), respectively. Median survival in *SOD1*-ALS patients with *SOD1* variants other than R116G, L145F and D91A (*n* = 38) was 248.0 months.

### Clinical phenotype in patients with homozygous and heterozygous D91A allele genotype

As homozygosity as well as heterozygosity of the pathogenic variant D91A has been related to ALS predisposition, clinical and demographic characteristics of these D91A patients were investigated, taking the number of alleles affected per patient into account (Table 3).

**Table 3:** Clinical features of ALS patients with homozygous and heterozygous *SOD1* D91A genotypes

	Homozygous (*n* = 6)	Heterozygous (*n* = 4)
**Age of onset** (mean, SD)	53.2 (± 12.7) (*n* = 6)	46.8 (± 15.5) (*n* = 4)
**Sex**		
Male	50.0% (*n* = 3)	25.0% (*n* = 1)
Female	50.0% (*n* = 3)	75.0% (*n* = 3)
**Onset**		
Spinal	100.0% (*n* = 3)	100.0% (*n* = 3)
Bulbar	0.0% (*n* = 0)	0.0% (*n* = 0)
**Family history**		
Positive	66.7% (*n* = 4)	25.0% (*n* = 1)
Negative	33.3% (*n* = 2)	75.0% (*n* = 3)
**ALSFRS-R**^**a**^ (1st visit) (median, IQR)	41.0 (38.0–44.0) (*n* = 4)	35.0 (27.0–37.0) (*n* = 4)
**Progression rate** (median, IQR) (onset to last visit)	0.04 (0.02–0.08) (*n* = 4)	0.03 (0.02–0.11) (*n* = 4)
**BMI**^**b**^** in kg/m**^**2**^ (mean, SD)	23.0 (± 2.4) (*n* = 2)	25.2 (± 5.3) (*n* = 4)
**Diagnostic delay in months** (mean, SD)	35.0 (± 31.2) (*n* = 3)	78.0 (± 17.0) (*n* = 3)

^a^*ALSFRS-R* ALS Functional Rating Scale-Revised

^b^*BMI* body mass index

Patients carrying a D91A on both alleles, i.e. in homozygous state (*n* = 6) all had a spinal onset of the disease, with a mean age of onset of 53.2 years (SD ± 12.7 years; *n* = 6), equal shares of males and females, but a positive family history of ALS in only 33.3%. ALS was diagnosed after a diagnostic delay of 35.0 months (mean, SD ± 31.2; *n* = 3). At first visit patients showed a median ALSFRS-R of 41.0 (IQR 38.0–44.0; *n* = 4), which declined in the median 0.04 points/month (IQR 0.02–0.08) until the last visit. Two patients with homozygous allele genotype died during the observation period after 141.0 and 198.0 months, respectively.

Age of onset in patients with heterozygous (*n* = 4) D91A allele genotype was 46.8 years (SD ± 15.5 years; *n* = 4). 75.0% (*n* = 3) of the included four patients were female and family history was also apparently negative in 75.0% (*n* = 3). All patients had a spinal onset of the disease. ALS was diagnosed after a mean delay of 78.0 months (SD ± 17.0 months; *n* = 3). ALSFRS-R was 35.0 (median, IQR 27.0–37.0; *n* = 4) at first visit and showed a median progression rate of 0.03 points lost/month (IQR 0.02–0.11; *n* = 4) until the last visit.

### Effect of tofersen treatment

In 10 patients receiving tofersen treatment in the German EAP, we compared demographic data and clinical outcome parameters, including ALSFRS-R and NfL serum levels during therapy (Table 4). At the start of tofersen treatment, age ranged between 54–61 years in patients with R116G, 52–67 years in patients with D91A, and 46–66 years in patients with L145F. Therapeutic delay was the shortest in R116G carriers with two patients receiving tofersen already six months after disease onset. However, one patient with R116G started tofersen therapy only after 110 months and therefore approximately nine years after disease onset. In patients with D91A, therapeutic delay ranged from 20–51 months with the earliest treatment in one of the patients with a homozygous allele genotype. Patients with L145F associated *SOD1*-ALS received tofersen treatment between 43–65 months after disease onset.

**Table 4:** Clinical characteristics of patients with R116G, D91A and L145F variants under tofersen treatment

*SOD1* mutation type	Age (years)	Delay (months)	ALSFRS-R (1st visit)	ALSFRS-R (last visit)	NfL (1st visit)	NfL (last visit)	Duration (months)	Visits (number)
R116G_1	59	17	35	32	81	29	11.5	14
R116G_2	55	6	38	20	186	78	7.7	10
R116G_3	54	110	45	45	177	28	10.3	13
R116G_4	61	6	42	42	21	17	1.9	4
D91A_1	61	51	32	35	37	18	11.4	14
D91A_2	67	40	32	33	78	22	11.6	14
D91A_3	52	20	44	43	58	36	8.9	11
L145F_1	46	65	18	22	35	18	12.1	15
L145F_2	51	43	38	37	113	64	3.1	5
L145F_3	66	51	27	30	41	26	2.8	5

*SOD1* superoxide dismutase 1, *age* age at first administration of tofersen, *delay* time between onset of ALS and tofersen treatment, *ALS* Functional Rating Scale-Revised, *NfL* neurofilament light chain (pg/ml), *duration* time of tofersen treatment

**Fig. 3:**
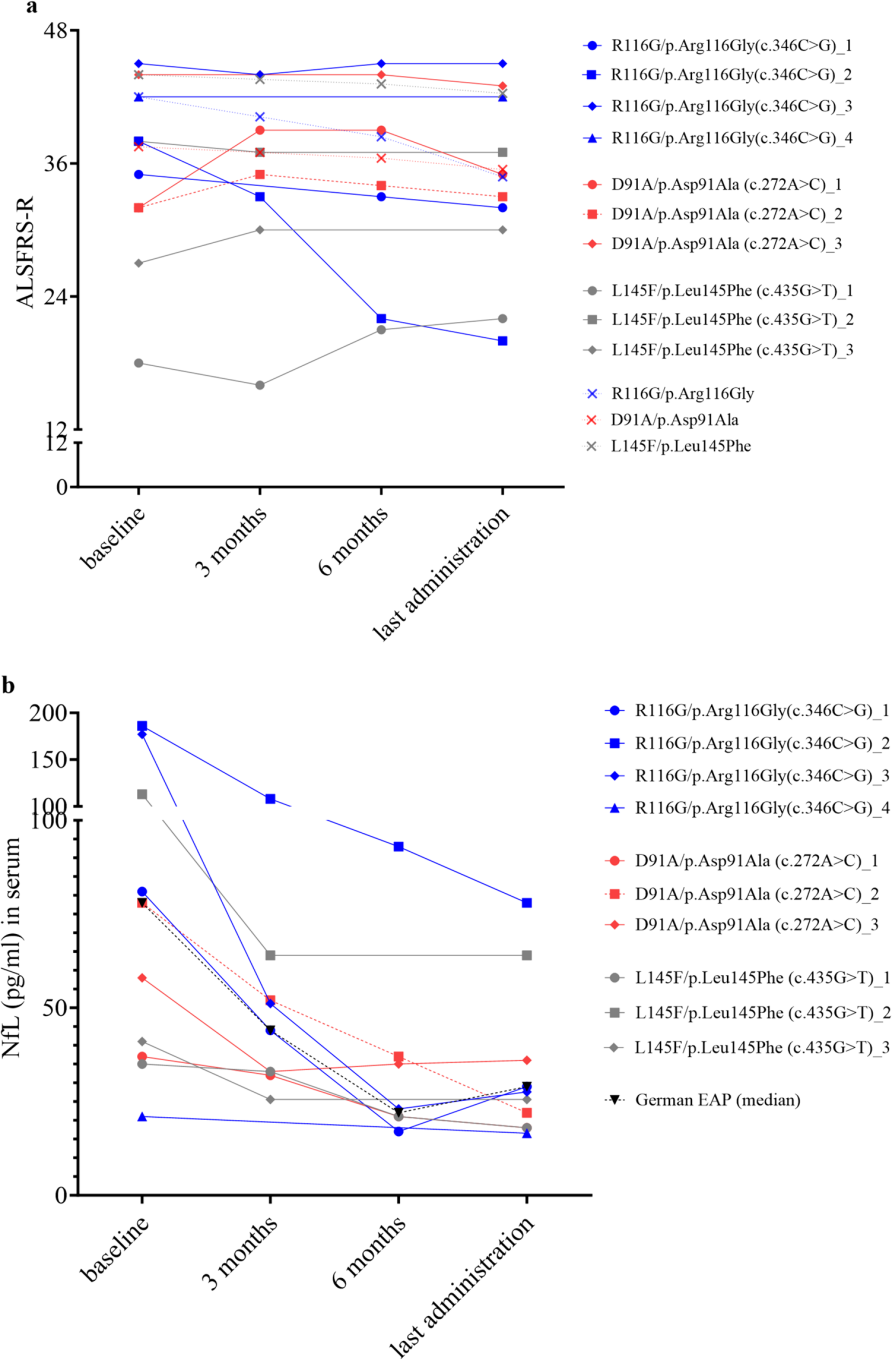
ALSFRS-R and neurofilaments on individual level depending on *SOD1* variants. Graphs show changes of ALSFRS-R and NfL levels (pg/ml) in serum prior to first administration of tofersen as well as after three and six months of therapy, and at last administration. (**a**) ALSFRS-R (**b**) NfL in serum (pg/ml). Time between first and last administration: R116G_1 11.5 months, R116G_2 7.7 months, R116G_3 10.3 months, R116G_4 1.9 months, D91A_1 11.4 months, D91A_2 11.6 months, D91A_3 8.9 months, L145F_1 12.1 months, L145F_2 3.1 months and L145F_3 2.8 months. Heterozygous allele genotype of D91A is shown in dashed lines. (**a**) ALSFRS-R after 3, 6, 9 months and 12 months (last visit) in patients with SOD1-ALS prior to initiation of the EAP is shown in x and dashed lines and was extrapolated from ALSFRS-R progression rate, calculated as median loss of points in ALSFRS-R/month between first and last visit, in patients with R116G (median time between first and last visit 8.0 months, IQR 5.5-27.0 months; n = 9), D91A (median 43.5 months, IQR 23.3-50.8 months; n = 6) and L145F (median 8.0 months, IQR 7.0–16.0 months; n = 3) until 2019. (**b**) Median NfL serum levels (pg/ml) of patients with SOD1-ALS from the German Early Access Program (EAP) are shown in a dashed black line (baseline n = 17, 3 months n = 17, 6 months n = 10, time between first and last administration median 6.0 months, IQR 2.5-10.5 months; n = 17). *ALSFRS-R* Amyotrophic lateral sclerosis functional rating scale revised, *NfL* neurofilament light chain (pg/ml).

During tofersen therapy, ALSFRS-R was stable in most of the participating patients (Fig. 3a). Only R116G_2 showed a fast decrease of ALSFRS-R in spite of a short therapeutic delay of 6 months, whereas an only slight disease progression was detected in R116G_1, D91A_3, and L145F_2. The ALSFRS-R remained unchanged in R116G_3 and R116G_4, whereas D91A_1, D91A_2, L145F_1, and L145F_3 even showed an increase of ALSFRS-R during the observation period.

All patients, independent of the *SOD1* variant, showed a reduction of NfL serum levels during tofersen treatment (Fig. 3b). This effect was also present in the patient with a heterozygous D91A allele genotype. Individual visits and treatment durations are depicted in Table 4.

**Fig. 4:**
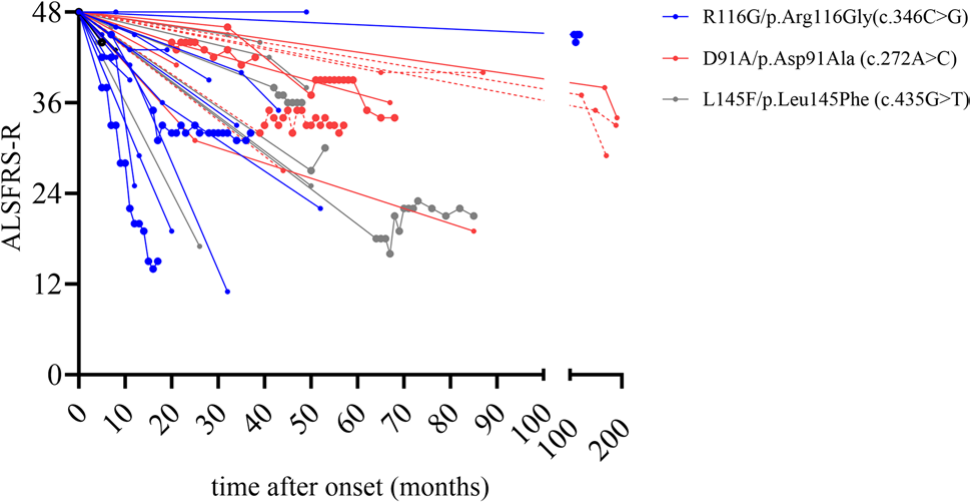
ALSFRS-R in R116G carriers, D91A carriers (homozygous and heterozygous) and L145F carriers on individual level. Graphs show ALSFRS-R values between onset of the disease and last visit. Retrospectively analyzed R116G carriers n = 14, R116G carriers treated with tofersen in the German Early Access Program (EAP) n = 4. Retrospectively analyzed D91A carriers n = 8 (n = 4 patients with homozygous and n = 4 patients with heterozygous allele genotype), D91A carriers treated with tofersen in the German EAP n = 3 (n = 2 patients with homozygous and n = 1 patient with heterozygous allele genotype). Heterozygous allele genotype of D91A is shown in dashed lines. Retrospectively analyzed L145F carriers n = 6, L145F carriers treated with tofersen in the German EAP n = 3. Patients treated with tofersen in the German EAP are highlighted with bold symbols, raw data of all patients is shown in Supplementary Table 1. *ALSFRS-R* Amyotrophic lateral sclerosis functional rating scale revised.

All available ALSFRS-R scores from disease onset to the last visit of retrospectively analyzed patients carrying a (likely) pathogenic R116G, D91A and L145F variant and of patients with these three variants who were treated with tofersen are shown separately (Fig. 4; Supplementary Table 1).

## Discussion

In this study, we analyzed and compared various clinical features of the most frequent (likely) pathogenic variants of *SOD1*-ALS in the German population, in order to characterize differences with regard to the clinical phenotype, which is highly relevant in the context of planning and evaluating new therapeutic approaches, such as tofersen.

Patients with *SOD1*-ALS carrying R116G, D91A, or L145F variants showed broad commonalities with regard to their clinical phenotypes, as well as demographic features like disease onset around the age of 50, spinal onset with about equal proximal and distal affection, and normal to slightly increased BMI. However, we also obtained some relevant differences, including survival, progression rate, and family history.

In accordance with the findings of Rabe et al. [15], patients with R116G variants showed a faster disease progression, including a significantly shorter survival, compared to patients carrying a D91A or L145F variant. Furthermore, ALSFRS-R progression rates were faster compared to patients with D91A, but still significantly slower compared to *C9orf72* or sporadic ALS [29]. Due to the faster disease progression, diagnostic delay of ALS in R116G patients was 10.0 months and therefore comparable to sporadic ALS [29]. On the other hand, comparatively long diagnostic delays of 57.5 months in D91A and 21.5 months in L145F carriers are likely explained by the slow disease progression rates associated with these variants. In accordance with the findings of Ruf et al.[8], who reported D91A to be the most frequent pathogenic variant in apparently sporadic cases of *SOD1*-ALS in Germany, 50% of patients carrying a D91A variant were sporadic, whereas almost all R116G and L145F carriers reported a positive family history of ALS. Negative family history in combination with slow progression rate might explain why D91A *SOD1*-ALS is only diagnosed at a median ALSFRS-R of 37.5, whereas patients with R116G or L145F had higher ALSFRS-R values at diagnosis. Especially in patients with D91A, this diagnostic delay might be problematic, as it is accompanied by an advanced loss of motor function and leads to a delayed initiation of tofersen therapy. In contrast to R116G and L145F carriers, more female patients carried a pathogenic D91A variant.

In our study, patients carrying the most common *SOD1* variants R116G (31% vs. 29% of cases), D91A (12% vs. 11% of cases), and L145F (7% vs. 11% of cases) [15] were detected with a similar frequency compared to previously reported results from a large German study including only patients with familial ALS [15]. Consistent descriptions of high frequencies of R116G in several studies of the German population [15, 30] can be explained by the hypothesis that they originate from a common founder and therefore share allele and haplotype characteristics [30]. Therefore, R116G seems to be the most relevant variant in German speaking countries, whereas none of the patients in this study showed an A5V mutation, which is the most common pathogenic variant in the United States [11, 13, 31]. Furthermore, D91A and L145F, but not R116G, variants were detected in a monocentric Italian study [32]; therefore, R116G seems to be most prevalent in Germany.

The special need for a clinical characterization of patients with *SOD1*-ALS in the German population becomes also evident by the fact that a useful web-tool, recently developed by Spargo et al.[33], which allows easy comparison of age of onset and disease duration among different *SOD1* variants, contains data of 1383 *SOD1*-ALS patients (A5V *n* = 312, D91A *n* = 83, L145F *n* = 69) [33], but only of one patient with R116G, which was found in 31.3% of the patients in our cohort.

In line with these data of a large international study, median age of onset in our patients was 50 years in D91A (vs. 49 years) and 54 years in L145F (vs. 53 years) carriers [34]. These pathogenic variants showed also a comparatively long disease duration [34]. However, diagnostic delay of 10 months in the general *SOD1*-ALS population corresponded to our results in patients carrying R116G variants [34].

Clinical manifestation of *SOD1*-ALS caused by the pathogenic variant D91A has been described to differ depending on whether the variant occurs on one or both alleles [35]. It is assumed, that families presenting with this variant as kind of a recessive trait, share a common founder as well as a protective factor closely linked to *SOD1*, which leads to lower penetrance with manifestation of ALS only in individuals with a homozygous allele genotype [35]. These patients show a uniform phenotype predominantly affecting the lower motoneurons and the lower limbs and have a longer disease duration [35]. Therefore we separately presented clinical and demographic characteristics of patients with D91A with a homozygous and a heterozygous allele genotype. All of the EAP patients showed a beneficial response to tofersen treatment as measured by NfL serum levels. As we could also detect a reduction of NfL in the D91A patient with a heterozygous allele genotype, our findings strengthen the hypothesis that alteration of *SOD1* is causative for the disease and not only an incidental finding. Furthermore, except one patient with R116G, who showed a fast progression in ALSFRS-R in spite of a comparatively short diagnostic delay of 6 months and a decrease in NfL levels during tofersen therapy, all included patients showed an almost stable course of the ALSFRS-R. Therefore, it can be concluded that all treated patients likely benefited from tofersen treatment, regardless of their *SOD1* variants. Nevertheless, it is necessary to re-evaluate these findings in a larger cohort after a prolonged period of time, and extend them to different *SOD1* variants.

We could observe a huge heterogeneity in ALSFRS-R progression rate in *SOD1*-ALS patients with and without tofersen therapy, even among carriers of the same pathogenic *SOD1* variant. Exogenous risk factors like physical activity [36], head trauma, smoking and alcohol consumption [37], occupational and environmental factors [38], reflected in regional differences in ALS incidence [39], as well as epigenetic factors [40, 41] could have a partial influence on this finding. Under tofersen therapy, these differences in the ALSFRS-R slope were not reflected in NfL levels, which showed a decline in all patients. This urges the need for the development of new surrogate biomarkers of progression rate to predict the clinical outcome of tofersen therapy. Since the causes of differences in treatment response, including single cases of non-responders in ALSFRS-R, are not understood yet, potentially contributing parameters such as alterations in SOD1 protein levels and SOD1 activity should be investigated in addition to the pathogenic variant.

A detailed knowledge of different clinical phenotypes of *SOD1*-ALS is relevant, as exemplified by a patient with L145F in our cohort. In this patient, diagnosis of ALS and, consequently, treatment with tofersen, was delayed by one year due to an atypical clinical presentation with onset in the lower limbs, prominent sensory deficits, and incontinence. These unusual features are in line with the findings of Marjanović et al. [42], who reported an onset in the lower extremities with sphincter dysfunction in 67% and sensory deficits in more than 50% of the Serbian L145F-*SOD1*-ALS population.

It is likely that our observations can also be transferred to other populations with a comparable *SOD1* variant spectrum, because it has been previously described that the same genotypes lead to comparable clinical phenotypes, even across different populations [14]. In line with this hypothesis, in the cohort of Marjanović et al. [42], who found D91A and L145F to be the most common pathogenic variants in the Serbian *SOD1*-ALS population, age of onset and spinal onset were similar compared to our cohort. Likewise, a typical clinical phenotype with onset in the lower extremities, slow progression rate and long survival has also been described for patients with homozygous D91A allele genotypes in Scandinavia [43].

Surprisingly, median survival in patients with pathogenic *SOD1* variants other than R116G, D91A and L145F was 248.0 months and therefore extremely long. Although this means a more benign course of the disease in these patients, it has to be discussed whether a selection bias could have contributed to this finding, as more slow-progressing patients were still alive when genetic testing for ALS became standard in the recent years. Due to the huge heterogeneity in ALSFRS-R progression rates even within carriers of the same pathogenic variant, which does not allow to predict the course of disease on an individual level at the time of diagnosis, tofersen therapy should be also recommended to these patients.

Our study is not without limitations. The tofersen analysis is limited by the small number of cases. Some information like manifestation of first paresis and, consequently survival as well as diagnostic and therapeutic delay, are largely based on anamnestic information and, therefore, are prone to bias. In general, due to small sample sizes, it was only possible to compare the most prevalent *SOD1* variants, which also applies to the comparison between patients with homozygous and heterozygous D91A genotypes. However, it would be interesting to systematically characterize less frequent pathogenic variants, which so far have only been described in case series [32, 44]. Since tofersen was shown to be an effective treatment for *SOD1*-ALS [21, 22], genetic testing for pathogenic *SOD1* variants should be recommended early to all ALS patients, regardless of their family history.

In summary, although patients with *SOD1*-ALS carrying R116G, D91A or L145F variants showed broad commonalities, we obtained some relevant differences, including a faster progression rate with shorter survival, but also shorter diagnostic delay in patients with R116G, and a comparatively benign disease course and high share of patients with negative family history in patients with D91A. In a small subgroup of ten patients, benefit from tofersen therapy, measured by ALSFRS-R and NfL serum levels, seemed to be independent of the *SOD1* variant. In conclusion, a profound knowledge of the clinical phenotypes associated with different pathogenic variants of *SOD1*-ALS will be important in the future in order to accelerate diagnosis and ensure early initiation of tofersen therapy.

## Supplementary Information

Below is the link to the electronic supplementary material.

Supplementary file1 (DOCX 26 KB)
