# Correction: Clinical characterization of common pathogenic variants of *SOD1*-ALS in Germany

**DOI:** 10.1007/s00415-025-12952-1

**Published:** 2025-03-08

**Authors:** Maximilian Wiesenfarth, Yalda Forouhideh-Wiesenfarth, Zeynep Elmas, Özlem Parlak, Ulrike Weiland, Christine Herrmann, Joachim Schuster, Axel Freischmidt, Kathrin Müller, Reiner Siebert, Kornelia Günther, Elke Fröhlich, Antje Knehr, Tatiana Simak, Franziska Bachhuber, Martin Regensburger, Susanne Petri, Thomas Klopstock, Peter Reilich, Florian Schöberl, Peggy Schumann, Peter Körtvélyessy, Thomas Meyer, Wolfgang P. Ruf, Simon Witzel, Hayrettin Tumani, David Brenner, Johannes Dorst, Albert C. Ludolph

**Affiliations:** 1 Department of Neurology, Ulm University, Oberer Eselsberg 45, 89081 Ulm, Germany; 2 German Centre for Neurodegenerative Diseases (DZNE) Site Ulm, 89081 Ulm, Germany; 3 Institute of Human Genetics, Ulm University and Ulm University Medical Center, 89081 Ulm, Germany; 4 Department of Molecular Neurology, Friedrich-Alexander-Universität Erlangen-Nürnberg (FAU), 91054 Erlangen, Germany; 5 Deutsches Zentrum Immuntherapie (DZI), University Hospital Erlangen, 91054 Erlangen, Germany; 6 Department of Neurology, Hannover Medical School, 30625 Hannover, Germany; 7 Department of Neurology with Friedrich-Baur-Institute, LMU University Hospital, LMU Munich, 80336 Munich, Germany; 8 German Centre for Neurodegenerative Diseases (DZNE) Site Munich, 81377 Munich, Germany; 9 Munich Cluster for Systems Neurology (SyNergy), 81377 Munich, Germany; 10 Ambulanzpartner Soziotechnologie GmbH, 13353 Berlin, Germany; 11 Department of Neurology, Center for ALS and other Motor Neuron Disorders, Charité—Universitätsmedizin Berlin, Corporate Member of Freie Universität Berlin, Humboldt-Universität zu Berlin, Berlin Institute of Health, 13353 Berlin, Germany; 12 German Centre for Neurodegenerative Diseases (DZNE) Site Magdeburg, 39120 Magdeburg, Germany

**Correction: Journal of Neurology (2024) 271:6667–6679** 10.1007/s00415-024-12564-1

In the original version of this article, the disease progression rates (ALSFRS-R between onset and last visit) were not reported in points lost per month as described in the text, but in points lost per week (in the text, Tables 2, 3 and Fig. 1b). This affects the progression rates in the text, Tables 2, 3 and Fig. 1b.

In the abstract, fifth sentence which previously read

Moreover, R116G patients had the fastest median ALSFRS-R progression rate with 0.12 (IQR 0.07–0.20) points lost per month.

Should have read

Moreover, R116G patients had the fastest median ALSFRS-R progression rate with 0.51 (IQR 0.28–0.87) points lost per month.

In the section “ALSFRS‑R, progression rate and diagnostic delay” first paragraph which previously read

Consistent with the survival data, *SOD1*-ALS patients with R116G variants showed a median disease progression rate of 0.12 ALSFRS-R points lost per month (IQR 0.07–0.20) between onset and last visit, which was more pronounced compared to patients with pathogenic D91A (0.03, IQR 0.02–0.08; *n* = 8; p = 0.02) and L145F variants (median 0.06, IQR 0.04–0.14; *n* = 6; p = 0.21), while D91A and L145F patients showed a quite similar, slow median progression rate (p = 0.35; Fig. 1b). Moreover, these differences in ALSFRS-R progression rate were already detected in an early phase of the disease, as a decline of 0.62 points (IQR 0.25–0.76; *n* = 14) per month in ALSFRS-R was found in patients with R116G variants compared to 0.16 (IQR 0.09–0.44; *n* = 8; p = 0.04) in patients with D91A and 0.38 with L145F variants (IQR 0.14–1.05; *n* = 6; p = 0.73) between the onset of the disease and the first visit at a MND reference center. In line with this faster disease progression, also the median delay of ALS diagnosis in patients carrying R116G variants was shorter (median 10.0 months, IQR 5.5–11.5; *n* = 13) than in patients with D91A (median 57.5 months, IQR 14.0–83.0; *n* = 6; p < 0.001) and in patients with L145F variants (median 21.5 months, IQR 5.8–38.8; *n* = 4; p = 0.27; Fig. 1c). Of note, median ALSFRS-R progression rates in patients with R116G, D91A and L145F were lower compared to patients carrying less frequent *SOD1* variants (0.50, IQR 0.13–1.38; *n* = 32; Table 2). Median ALSFRS-R at first visit was 42.5 (IQR 39.0–45.0; *n* = 14) in patients with R116G and therefore higher compared to 37.5 (IQR 32.0–40.8; *n* = 8) in D91A (p = 0.04; Fig. 1d) and similar to L145F carriers (43.0, IQR 23.0–45.3; *n* = 6; p = 0.83; Fig. 1d).

Should have read

Consistent with the survival data, *SOD1*-ALS patients with R116G variants showed a median disease progression rate of 0.51 ALSFRS-R points lost per month (IQR 0.28–0.87) between onset and last visit, which was more pronounced compared to patients with pathogenic D91A (0.14, IQR 0.08–0.33; *n* = 8; p = 0.02) and L145F variants (median 0.27, IQR 0.17–0.62; *n* = 6; p = 0.24), while D91A and L145F patients showed a quite similar, slow median progression rate (p = 0.28; Fig. 1b). Moreover, these differences in ALSFRS-R progression rate were already detected in an early phase of the disease, as a decline of 0.62 points (IQR 0.25–0.76; *n* = 14) per month in ALSFRS-R was found in patients with R116G variants compared to 0.16 (IQR 0.09–0.44; *n* = 8; p = 0.04) in patients with D91A and 0.38 with L145F variants (IQR 0.14–1.05; *n* = 6; p = 0.73) between the onset of the disease and the first visit at a MND reference center. In line with this faster disease progression, also the median delay of ALS diagnosis in patients carrying R116G variants was shorter (median 10.0 months, IQR 5.5–11.5; *n* = 13) than in patients with D91A (median 57.5 months, IQR 14.0–83.0; *n* = 6; p < 0.001) and in patients with L145F variants (median 21.5 months, IQR 5.8–38.8; *n* = 4; p = 0.27; Fig. 1c). Of note, median ALSFRS-R progression rates in patients with D91A and L145F were lower compared to patients carrying less frequent *SOD1* variants (0.50, IQR 0.13–1.38; *n* = 32; Table 2). Median ALSFRS-R at first visit was 42.5 (IQR 39.0–45.0; *n* = 14) in patients with R116G and therefore higher compared to 37.5 (IQR 32.0–40.8; *n* = 8) in D91A (p = 0.04; Fig. 1d) and similar to L145F carriers (43.0, IQR 23.0–45.3; *n* = 6; p = 0.83; Fig. 1d).

In the section “Clinical phenotype in patients with homozygous and heterozygous D91A allele genotype” second and third paragraph which previously read

Patients carrying a D91A on both alleles, i.e. in homozygous state (*n* = 6) all had a spinal onset of the disease, with a mean age of onset of 53.2 years (SD ± 12.7 years; *n* = 6), equal shares of males and females, but a positive family history of ALS in only 33.3%. ALS was diagnosed after a diagnostic delay of 35.0 months (mean, SD ± 31.2; *n* = 3). At first visit patients showed a median ALSFRS-R of 41.0 (IQR 38.0–44.0; *n* = 4), which declined in the median 0.04 points/month (IQR 0.02–0.08) until the last visit. Two patients with homozygous allele genotype died during the observation period after 141.0 and 198.0 months, respectively.

Age of onset in patients with heterozygous (*n* = 4) D91A allele genotype was 46.8 years (SD ± 15.5 years; *n* = 4). 75.0% (*n* = 3) of the included four patients were female and family history was also apparently negative in 75.0% (*n* = 3). All patients had a spinal onset of the disease. ALS was diagnosed after a mean delay of 78.0 months (SD ± 17.0 months; *n* = 3). ALSFRS-R was 35.0 (median, IQR 27.0–37.0; *n* = 4) at first visit and showed a median progression rate of 0.03 points lost/month (IQR 0.02–0.11; *n* = 4) until the last visit.

Should have read

Patients carrying a D91A on both alleles, i.e. in homozygous state (*n* = 6) all had a spinal onset of the disease, with a mean age of onset of 53.2 years (SD ± 12.7 years; *n* = 6), equal shares of males and females, but a positive family history of ALS in only 33.3%. ALS was diagnosed after a diagnostic delay of 35.0 months (mean, SD ± 31.2; *n* = 3). At first visit patients showed a median ALSFRS-R of 41.0 (IQR 38.0–44.0; *n* = 4), which declined in the median 0.17 points/month (IQR 0.07–0.33) between onset and the last visit. Two patients with homozygous allele genotype died during the observation period after 141.0 and 198.0 months, respectively.

Age of onset in patients with heterozygous (*n* = 4) D91A allele genotype was 46.8 years (SD ± 15.5 years; *n* = 4). 75.0% (*n* = 3) of the included four patients were female and family history was also apparently negative in 75.0% (*n* = 3). All patients had a spinal onset of the disease. ALS was diagnosed after a mean delay of 78.0 months (SD ± 17.0 months; *n* = 3). ALSFRS-R was 35.0 (median, IQR 27.0–37.0; *n* = 4) at first visit and showed a median progression rate of 0.11 points lost/month (IQR 0.08–0.46; *n* = 4) between onset and the last visit.

Tables 2 and 3 which previously read

**Table 2** Clinical features of patients with different variants of the *SOD1* gene
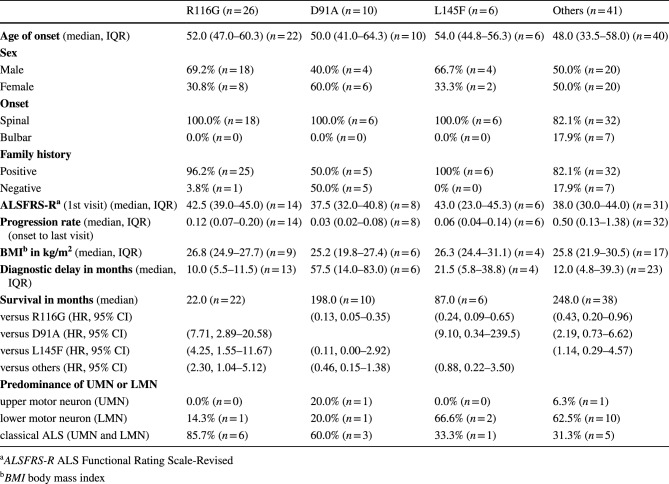


**Table 3** Clinical features of ALS patients with homozygous and heterozygous *SOD1* D91A genotypes
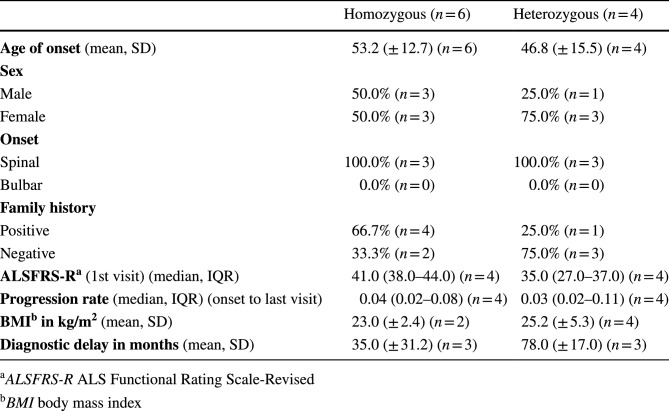


Tables 2 and 3 should have read

**Table 2:** Clinical features of patients with different variants of the *SOD1* gene

	R116G (*n* = 26)	D91A (*n* = 10)	L145F (*n* = 6)	Others (*n* = 41)
**Age of onset** (median, IQR)	52.0 (47.0–60.3) (*n* = 22)	50.0 (41.0–64.3) (*n* = 10)	54.0 (44.8–56.3) (*n* = 6)	48.0 (33.5–58.0) (*n* = 40)
**Sex**				
Male	69.2% (*n* = 18)	40.0% (*n* = 4)	66.7% (*n* = 4)	50.0% (*n* = 20)
Female	30.8% (*n* = 8)	60.0% (*n* = 6)	33.3% (*n* = 2)	50.0% (*n* = 20)
**Onset**				
Spinal	100.0% (*n* = 18)	100.0% (*n* = 6)	100.0% (*n* = 6)	82.1% (*n* = 32)
Bulbar	0.0% (*n* = 0)	0.0% (*n* = 0)	0.0% (*n* = 0)	17.9% (*n* = 7)
**Family history**				
Positive	96.2% (*n* = 25)	50.0% (*n* = 5)	100% (*n* = 6)	82.1% (*n* = 32)
Negative	3.8% (*n* = 1)	50.0% (*n* = 5)	0% (*n* = 0)	17.9% (*n* = 7)
**ALSFRS-R** (1st visit) (median, IQR)	42.5 (39.0–45.0) (*n* = 14)	37.5 (32.0–40.8) (*n* = 8)	43.0 (23.0–45.3) (*n* = 6)	38.0 (30.0–44.0) (*n* = 31)
**Progression rate** (median, IQR) (onset to last visit)	0.51 (0.28–0.87) (*n* = 14)	0.14 (0.08–0.33) (*n* = 8)	0.27 (0.17–0.62) (*n* = 6)	0.50 (0.13–1.38) (*n* = 32)
**BMI in kg/m**^**2**^ (median, IQR)	26.8 (24.9–27.7) (*n* = 9)	25.2 (19.8–27.4) (*n* = 6)	26.3 (24.4–31.1) (*n* = 4)	25.8 (21.9–30.5) (*n* = 17)
**Diagnostic delay in months** (median, IQR)	10.0 (5.5–11.5) (*n* = 13)	57.5 (14.0–83.0) (*n* = 6)	21.5 (5.8–38.8) (*n* = 4)	12.0 (4.8–39.3) (*n* = 23)
**Survival in months** (median)	22.0 (*n* = 22)	198.0 (*n* = 10)	87.0 (*n* = 6)	248.0 (*n* = 38)
Versus R116G (HR, 95% CI)		(0.13, 0.05–0.35)	(0.24, 0.09–0.65)	(0.43, 0.20–0.96)
Versus D91A (HR, 95% CI)	(7.71, 2.89–20.58)		(9.10, 0.34–239.5)	(2.19, 0.73–6.62)
Versus L145F (HR, 95% CI)	(4.25, 1.55–11.67)	(0.11, 0.00–2.92)		(1.14, 0.29–4.57)
Versus others (HR, 95% CI)	(2.30, 1.04–5.12)	(0.46, 0.15–1.38)	(0.88, 0.22–3.50)	
**Predominance of UMN or LMN**				
Upper motor neuron (UMN)	0.0% (*n* = 0)	20.0% (*n* = 1)	0.0% (*n* = 0)	6.3% (*n* = 1)
Lower motor neuron (LMN)	14.3% (*n* = 1)	20.0% (*n* = 1)	66.6% (*n* = 2)	62.5% (*n* = 10)
Classical ALS (UMN and LMN)	85.7% (*n* = 6)	60.0% (*n* = 3)	33.3% (*n* = 1)	31.3% (*n* = 5)

*ALSFRS-R* ALS Functional Rating Scale-Revised, *BMI* body mass index

**Table 3:** Clinical features of ALS patients with homozygous and heterozygous *SOD1* D91A genotypes

	Homozygous (*n* = 6)	Heterozygous (*n* = 4)
**Age of onset** (mean, SD)	53.2 (± 12.7) (*n* = 6)	46.8 (± 15.5) (*n* = 4)
**Sex**		
Male	50.0% (*n* = 3)	25.0% (*n* = 1)
Female	50.0% (*n* = 3)	75.0% (*n* = 3)
**Onset**		
Spinal	100.0% (*n* = 3)	100.0% (*n* = 3)
Bulbar	0.0% (*n* = 0)	0.0% (*n* = 0)
**Family history**		
Positive	66.7% (*n* = 4)	25.0% (*n* = 1)
Negative	33.3% (*n* = 2)	75.0% (*n* = 3)
**ALSFRS-R** (1st visit) (median, IQR)	41.0 (38.0–44.0) (*n* = 4)	35.0 (27.0–37.0) (*n* = 4)
**Progression rate** (median, IQR) (onset to last visit)	0.17 (0.07–0.33) (*n* = 4)	0.11 (0.08–0.46) (*n* = 4)
**BMI in kg/m**^**2**^ (mean, SD)	23.0 (± 2.4) (*n* = 2)	25.2 (± 5.3) (*n* = 4)
**Diagnostic delay in months** (mean, SD)	35.0 (± 31.2) (*n* = 3)	78.0 (± 17.0) (*n* = 3)

*ALSFRS-R* ALS Functional Rating Scale-Revised, *BMI* body mass index

Figure 1 which previously appeared as
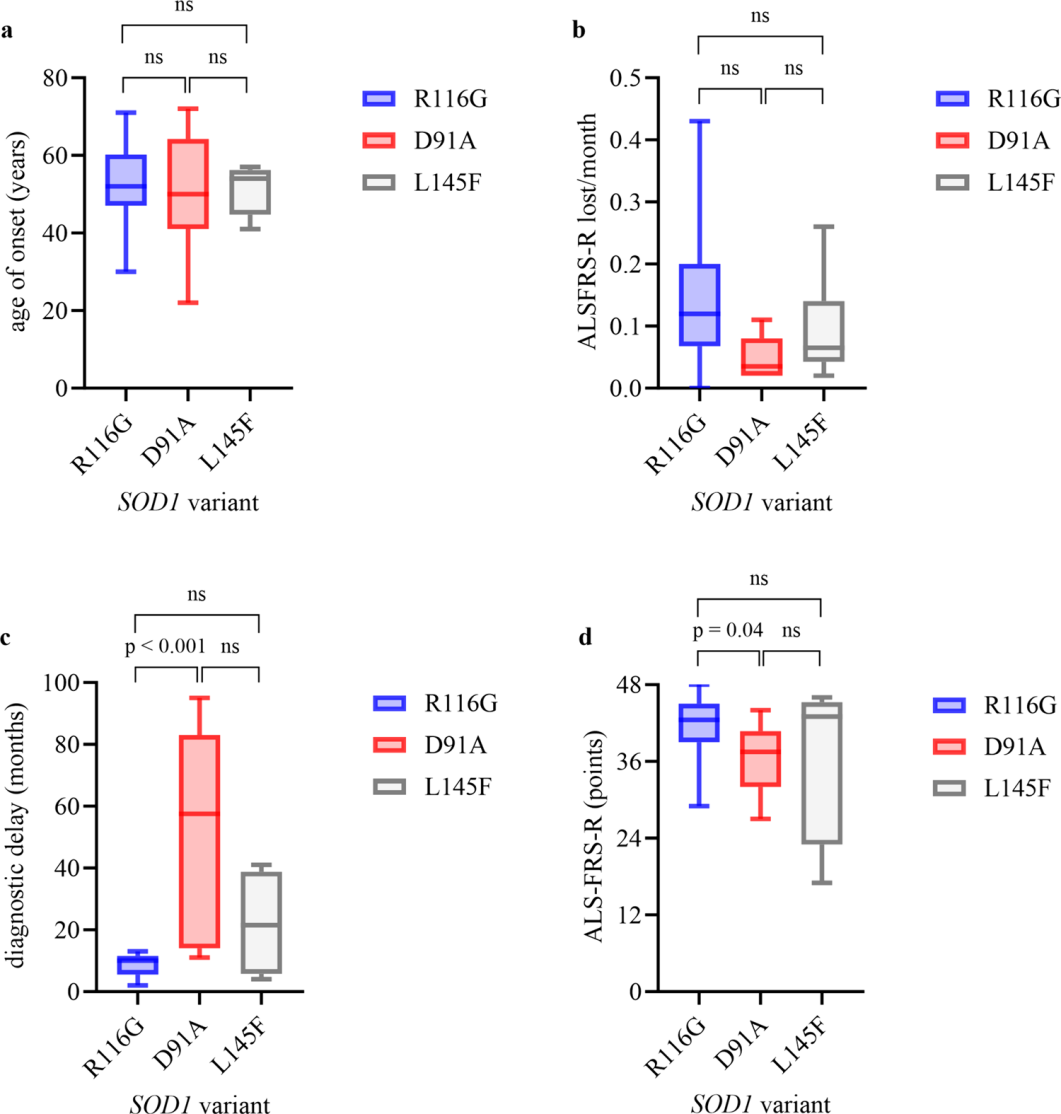


**Fig. 1** Clinical characteristics in R116G carriers vs. D91A carriers (homozygous and heterozygous) vs. L145F carriers. Boxplots show median (IQR; minimum–maximum). (**a**) age of onset (**b**) progression rate (**c**) diagnostic delay (**d**) ALSFRS-R at first visit. Experimental units *n* = number (**a**) R116G *n* = 22, D91A* n* = 10, p = 0.6814, R116G *n* = 22, L145F* n* = 6, p = 0.9239, D91A* n* = 10, L144F* n* = 6, p = 0.9798 (**b**) R116G* n* = 14, D91A* n* = 8, p = 0.0183, R116G* n* = 14, L145F* n* = 6, p = 0.2125, D91A* n* = 8, L145F* n* = 6, p = 0.3526 (**c**) R116G* n* = 13, D91A *n* = 6, p = 0.0008, R116G* n* = 13, L145F *n* = 4, p = 0.2723, D91A *n* = 6, L145F *n* = 4, p = 0.1286 (**d**) R116G* n* = 14, D91A *n* = 8, p = 0.0439, R116G* n* = 14, L145F* n* = 6, p = 0.08256, D91A* n* = 8, L145F* n* = 6, p = 0.4336. Mann–Whitney U test was used for two group comparison. A *P*-value of ≤ 0.05 was regarded as statistically significant. *ALSFRS-R* Amyotrophic lateral sclerosis functional rating scale revised

Figure 1 should have appeared as shown below

**Fig. 1:**
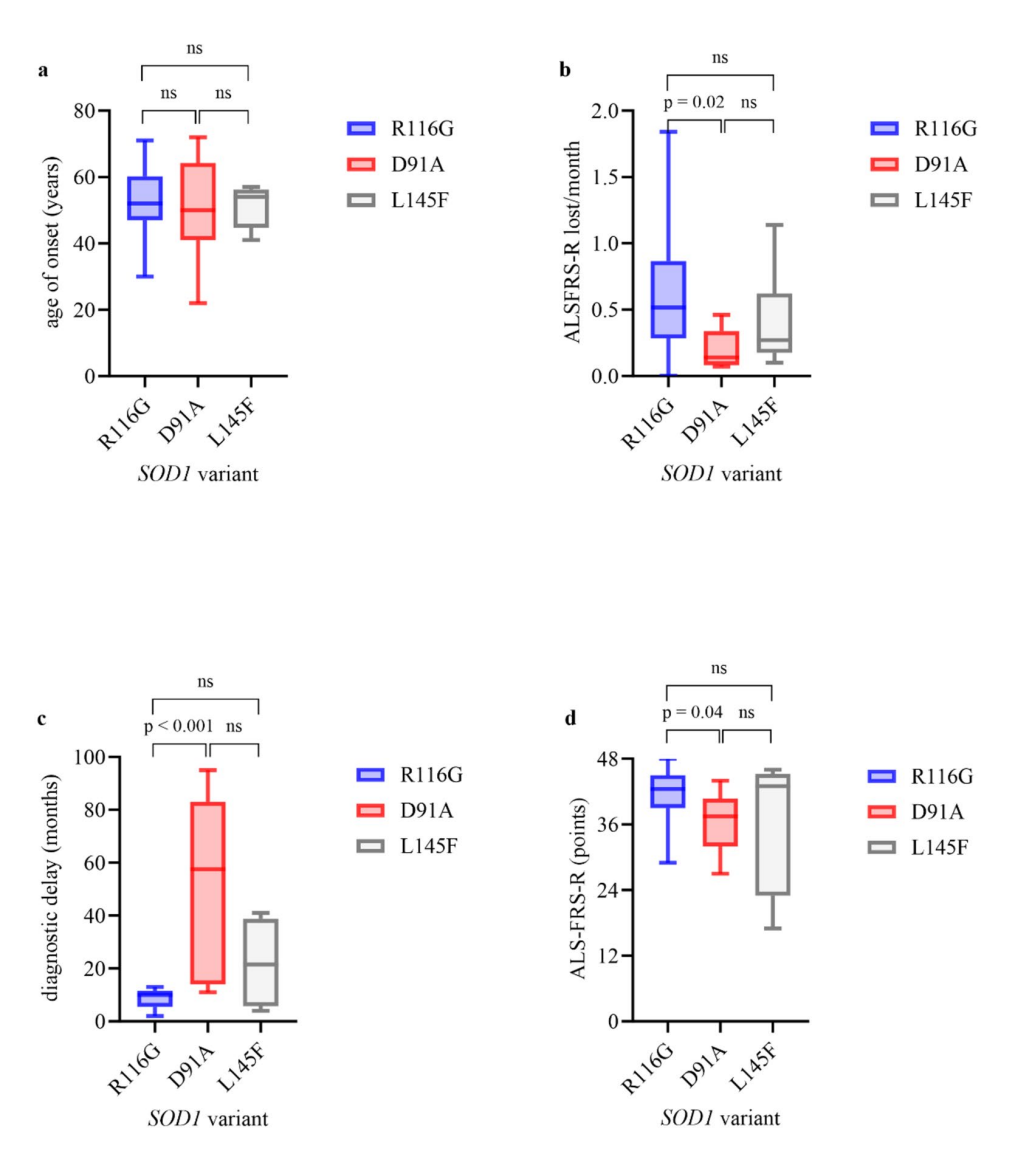
Clinical characteristics in R116G carriers vs. D91A carriers (homozygous and heterozygous) vs. L145F carriers. Boxplots show median (IQR; minimum–maximum). (**a**) Age of onset, (**b**) progression rate, (**c**) diagnostic delay, (**d**) ALSFRS-R at first visit. Experimental units *n* = number (**a**) R116G *n* = 22, D91A* n* = 10, p = 0.6814, R116G *n* = 22, L145F* n* = 6, p = 0.9239, D91A* n* = 10, L144F* n* = 6, p = 0.9798; (**b**) R116G* n* = 14, D91A* n* = 8, p = 0.0159, R116G* n* = 14, L145F* n* = 6, p = 0.2391, D91A* n* = 8, L145F* n* = 6, p = 0.2824; **c** R116G* n* = 13, D91A *n* = 6, p = 0.0008, R116G* n* = 13, L145F *n* = 4, p = 0.2723, D91A *n* = 6, L145F *n* = 4, p = 0.1286; (**d**) R116G* n* = 14, D91A* n* = 8, p = 0.0439, R116G* n* = 14, L145F* n* = 6, p = 0.08256, D91A* n* = 8, L145F* n* = 6, p = 0.4336. Mann–Whitney U test was used for two group comparison. A *P*-value of ≤ 0.05 was regarded as statistically significant. *ALSFRS-R* amyotrophic lateral sclerosis Functional Rating Scale Revised

