# Supplementary material for: Clinical characterization of common pathogenic variants of SOD1-ALS in Germany
Source: J Neurol. 2024 Aug 14;271(10):6667–79. doi: 10.1007/s00415-024-12564-1 (PMC11446975; doi:10.1007/s00415-024-12564-1)
Supplement: Supplementary file 1 — Supplementary file1 (DOCX 26 KB) [file 415_2024_12564_MOESM1_ESM.docx]

**Supplemetary table 1: Raw data of ALSFRS-R in R116G carriers, D91A carriers (homozygous and heterozygous) and L145F carriers.**

|  | **ALSFRS-R** | | | | | | | | | | | | | | | | | | | | | | | | | | | | | | | | | | | | | |
| --- | --- | --- | --- | --- | --- | --- | --- | --- | --- | --- | --- | --- | --- | --- | --- | --- | --- | --- | --- | --- | --- | --- | --- | --- | --- | --- | --- | --- | --- | --- | --- | --- | --- | --- | --- | --- | --- | --- |
| time after onset (months) | R116G | | | | | | | | | | | | | | **R116G** | | | | D91A | | | | *D91A* | | | | **D91A** | | ***D91A*** | L145F | | | | | | **L145F** | | |
| 0 | 48 | 48 | 48 | 48 | 48 | 48 | 48 | 48 | 48 | 48 | 48 | 48 | 48 | 48 | 48 | 48 | 48 | 48 | 48 | 48 | 48 | 48 | 48 | 48 | 48 | 48 | 48 | 48 | 48 | 48 | 48 | 48 | 48 | 48 | 48 | 48 | 48 | 48 |
| 4 |  |  |  |  |  |  |  |  |  |  |  |  |  |  |  |  |  |  |  |  |  |  |  |  |  |  |  |  |  |  | 44 |  |  |  |  |  |  |  |
| 5 |  |  |  |  |  | 44 |  |  |  |  | 45 |  |  |  |  | **38** |  | **42** |  |  |  |  |  |  |  |  |  |  |  |  |  |  |  |  |  |  |  |  |
| 6 |  |  |  |  |  |  |  |  | 42 |  |  |  |  |  |  | **38** |  |  |  |  |  |  |  |  |  |  |  |  |  |  |  |  |  |  |  |  |  |  |
| 7 |  |  |  |  |  |  |  |  |  |  |  |  |  |  | 45 | **33** |  | **42** |  |  |  |  |  |  |  |  |  |  |  | 46 |  |  |  |  |  |  |  |  |
| 8 |  |  | 48 | 46 | 43 |  |  |  |  |  |  |  | 42 |  |  | **33** |  |  |  |  |  |  |  |  |  |  |  |  |  |  |  |  |  |  |  |  |  |  |
| 9 |  |  |  |  |  |  |  |  |  |  |  |  |  |  |  | **28** |  |  |  |  |  |  |  |  |  |  |  |  |  |  |  |  |  |  |  |  |  |  |
| 10 |  |  |  |  |  |  |  |  |  |  |  |  |  |  |  | **28** |  |  |  |  |  |  |  |  |  |  |  |  |  |  |  |  |  |  |  |  |  |  |
| 11 | 41 | 43 |  |  |  |  |  |  | 39 |  |  |  |  |  |  | **22** |  |  |  |  |  |  |  |  |  |  |  |  |  |  |  |  |  |  |  |  |  |  |
| 12 |  |  |  |  |  |  |  | 45 |  |  |  |  | 25 |  |  | **20** |  |  |  |  |  |  |  |  |  |  |  |  |  |  |  |  |  |  |  |  |  |  |
| 13 |  |  |  |  |  |  |  |  |  |  |  |  |  | 29 |  | **20** |  |  |  |  |  |  |  |  |  |  |  |  |  |  |  |  |  |  |  |  |  |  |
| 14 |  |  |  |  |  |  |  |  |  |  |  |  |  |  |  | **19** |  |  |  |  |  |  |  |  |  |  |  |  |  |  |  |  |  |  |  |  |  |  |
| 15 |  |  |  |  |  |  |  |  |  |  |  |  |  |  |  | **15** |  |  |  |  |  |  |  |  |  |  |  |  |  |  |  |  |  |  |  |  |  |  |
| 16 |  |  |  |  |  |  |  |  |  |  |  |  |  |  | **35** | **14** |  |  |  |  |  |  |  |  |  |  |  |  |  |  |  |  |  |  |  |  |  |  |
| 17 |  |  |  |  |  |  |  |  |  |  |  |  |  |  | **31** | **15** |  |  |  |  |  |  |  |  |  |  |  |  |  |  |  |  |  |  |  |  |  |  |
| 18 |  |  |  |  |  |  |  |  |  | 36 |  |  |  |  | **33** |  |  |  |  |  |  |  |  |  |  |  |  |  |  |  |  |  |  |  |  |  |  |  |
| 19 |  | 43 |  |  |  |  |  |  |  |  |  |  |  |  |  |  |  |  |  |  |  |  |  |  |  |  |  |  |  |  |  |  |  |  |  |  |  |  |
| 20 |  |  |  |  |  |  |  |  |  |  |  |  |  | 19 | **32** |  |  |  |  |  | 44 |  |  |  |  |  | **44** |  |  |  |  |  |  |  |  |  |  |  |
| 21 |  |  |  |  |  |  |  |  |  |  |  |  |  |  | **32** |  |  |  |  |  |  | 41 |  |  |  |  | **43** |  |  |  |  |  |  |  |  |  |  |  |
| 22 |  |  |  |  |  |  |  |  |  |  |  |  |  |  | **33** |  |  |  |  |  |  |  |  |  |  |  | **44** |  |  |  |  |  |  |  |  |  |  |  |
| 23 |  |  |  |  |  |  |  |  |  |  |  |  |  |  | **32** |  |  |  |  |  |  |  |  |  |  |  | **44** |  |  |  |  |  |  |  |  |  |  |  |
| 24 |  |  |  |  |  |  |  |  |  |  |  |  |  |  |  |  |  |  |  |  |  |  |  |  |  |  | **44** |  |  |  |  |  |  |  |  |  |  |  |
| 25 |  |  |  |  |  |  |  |  |  |  |  |  |  |  | **33** |  |  |  |  | 31 |  |  |  |  |  |  | **44** |  |  |  |  |  |  |  |  |  |  |  |
| 26 |  |  |  |  |  |  |  |  |  |  |  |  |  |  | **32** |  |  |  |  |  |  |  |  |  |  |  |  |  |  |  |  |  |  | 17 |  |  |  |  |
| 27 |  |  |  |  |  |  |  |  |  |  |  |  |  |  |  |  |  |  |  |  |  |  |  |  |  |  | **43** |  |  |  |  |  |  |  |  |  |  |  |
| 28 |  |  |  |  |  |  |  | 39 |  |  |  |  |  |  | **32** |  |  |  |  |  |  |  |  |  |  |  |  |  |  |  |  |  |  |  |  |  |  |  |
| 29 |  |  |  |  |  |  |  |  |  |  |  |  |  |  | **32** |  |  |  |  |  |  |  |  |  |  |  | **42** |  |  |  |  |  |  |  |  |  |  |  |
| 30 |  |  |  |  |  |  |  |  |  |  |  |  |  |  | **32** |  |  |  |  |  |  |  |  |  |  |  |  |  |  |  |  |  |  |  |  |  |  |  |
| 31 |  |  |  |  |  |  |  |  |  |  |  |  |  |  | **32** |  |  |  |  |  |  |  |  |  |  |  |  |  |  |  |  |  |  |  |  |  |  |  |
| 32 | 11 |  |  |  |  |  |  |  |  |  |  |  |  |  | **32** |  |  |  |  |  |  |  |  |  |  |  | **43** | 46 |  |  |  |  | 45 |  |  |  |  |  |
| 34 |  |  |  |  |  |  | 33 |  |  |  |  |  |  |  | **31** |  |  |  |  |  |  |  |  |  |  |  |  |  |  |  |  |  |  |  |  |  |  |  |
| 35 |  |  |  |  |  |  |  |  |  |  |  | 40 |  |  |  |  |  |  |  |  |  |  |  |  |  |  | **41** |  |  |  |  |  |  |  |  |  |  |  |
| 36 |  |  |  |  |  |  |  |  |  |  |  |  |  |  | **31** |  |  |  |  |  |  |  |  |  |  |  |  |  |  |  |  |  |  |  |  |  |  |  |
| 37 |  |  |  |  |  |  |  |  |  |  |  |  |  |  | **32** |  |  |  |  |  |  |  |  |  |  |  |  |  |  |  |  |  |  |  |  |  |  |  |
| 38 |  |  |  |  |  |  |  |  |  |  |  |  |  |  |  |  |  |  |  |  |  |  |  |  |  |  | **42** |  |  |  |  |  |  |  |  |  |  |  |
| 39 |  |  |  |  |  |  |  |  |  |  |  |  |  |  |  |  |  |  |  |  |  |  |  |  |  |  |  |  | **32** |  |  |  | 44 |  |  |  |  |  |
| 40 |  |  |  |  |  |  |  |  |  |  |  |  |  |  |  |  |  |  |  |  |  |  |  |  |  |  |  |  | **33** |  |  |  |  |  |  |  |  |  |
| 41 |  |  |  |  |  |  |  |  |  |  |  |  |  |  |  |  |  |  |  |  |  |  |  |  |  |  |  |  | **35** |  |  | 42 |  |  |  |  |  |  |
| 42 |  |  |  |  |  |  |  |  |  |  |  |  |  |  |  |  |  |  |  |  |  |  |  |  |  |  |  |  | **34** |  |  |  |  |  |  |  | **38** |  |
| 43 |  |  |  |  |  |  |  |  |  |  |  | 35 |  |  |  |  |  |  |  |  |  |  |  |  |  |  |  |  | **33** |  |  |  |  |  |  |  | **37** |  |
| 44 |  |  |  |  |  |  |  |  |  |  |  |  |  |  |  |  |  |  |  |  |  |  |  | 27 |  |  |  |  | **34** |  |  |  |  |  |  |  | **37** |  |
| 45 |  |  |  |  |  |  |  |  |  |  |  |  |  |  |  |  |  |  |  |  |  |  |  |  |  |  |  |  | **35** |  |  |  |  |  |  |  | **36** |  |
| 46 |  |  |  |  |  |  |  |  |  |  |  |  |  |  |  |  |  |  |  |  |  |  |  |  |  |  |  |  | **32** |  |  |  |  |  |  |  | **36** |  |
| 47 |  |  |  |  |  |  |  |  |  |  |  |  |  |  |  |  |  |  |  |  |  |  |  |  |  |  |  |  | **35** |  |  |  |  |  |  |  | **36** |  |
| 48 |  |  |  |  |  |  |  |  |  |  |  |  |  |  |  |  |  |  |  |  |  |  |  |  |  |  |  |  | **35** |  |  |  |  |  |  |  | **36** |  |
| 49 |  |  | 48 |  |  |  |  |  |  |  |  |  |  |  |  |  |  |  |  |  |  |  |  |  |  |  |  |  | **33** |  |  | 38 |  |  |  |  |  |  |
| 50 |  |  |  |  |  |  |  |  |  |  |  |  |  |  |  |  |  |  |  |  |  |  |  |  |  |  |  | **37** | **33** |  |  |  |  |  | 25 |  |  | **27** |
| 51 |  |  |  |  |  |  |  |  |  |  |  |  |  |  |  |  |  |  |  |  |  |  |  |  |  |  |  | **39** | **34** |  |  |  |  |  |  |  |  |  |
| 52 |  |  |  |  |  |  |  |  |  | 22 |  |  |  |  |  |  |  |  |  |  |  |  |  |  |  |  |  | **39** | **33** |  |  |  |  |  |  |  |  |  |
| 53 |  |  |  |  |  |  |  |  |  |  |  |  |  |  |  |  |  |  |  |  |  |  |  |  |  |  |  | **39** | **34** |  |  |  |  |  |  |  |  | **30** |
| 54 |  |  |  |  |  |  |  |  |  |  |  |  |  |  |  |  |  |  |  |  |  |  |  |  |  |  |  | **39** | **33** |  |  |  |  |  |  |  |  |  |
| 55 |  |  |  |  |  |  |  |  |  |  |  |  |  |  |  |  |  |  |  |  |  |  |  |  |  |  |  | **39** | **33** |  |  |  |  |  |  |  |  |  |
| 56 |  |  |  |  |  |  |  |  |  |  |  |  |  |  |  |  |  |  |  |  |  |  |  |  |  |  |  | **39** | **32** |  |  |  |  |  |  |  |  |  |
| 57 |  |  |  |  |  |  |  |  |  |  |  |  |  |  |  |  |  |  |  |  |  |  |  |  |  |  |  | **39** | **33** |  |  |  |  |  |  |  |  |  |
| 58 |  |  |  |  |  |  |  |  |  |  |  |  |  |  |  |  |  |  |  |  |  |  |  |  |  |  |  | **39** |  |  |  |  |  |  |  |  |  |  |
| 59 |  |  |  |  |  |  |  |  |  |  |  |  |  |  |  |  |  |  |  |  |  |  |  |  |  |  |  | **39** |  |  |  |  |  |  |  |  |  |  |
| 62 |  |  |  |  |  |  |  |  |  |  |  |  |  |  |  |  |  |  |  |  |  |  |  |  |  |  |  | **35** |  |  |  |  |  |  |  |  |  |  |
| 64 |  |  |  |  |  |  |  |  |  |  |  |  |  |  |  |  |  |  |  |  |  |  |  |  |  |  |  |  |  |  |  |  |  |  |  | **18** |  |  |
| 65 |  |  |  |  |  |  |  |  |  |  |  |  |  |  |  |  |  |  |  |  |  |  | 40 |  |  |  |  | **34** |  |  |  |  |  |  |  | **18** |  |  |
| 66 |  |  |  |  |  |  |  |  |  |  |  |  |  |  |  |  |  |  |  |  |  |  |  |  |  |  |  |  |  |  |  |  |  |  |  | **18** |  |  |
| 67 |  |  |  |  |  |  |  |  |  |  |  |  |  |  |  |  |  |  |  |  | 36 |  |  |  |  |  |  |  |  |  |  |  |  |  |  | **16** |  |  |
| 68 |  |  |  |  |  |  |  |  |  |  |  |  |  |  |  |  |  |  |  |  |  |  |  |  |  |  |  | **34** |  |  |  |  |  |  |  | **21** |  |  |
| 69 |  |  |  |  |  |  |  |  |  |  |  |  |  |  |  |  |  |  |  |  |  |  |  |  |  |  |  |  |  |  |  |  |  |  |  | **19** |  |  |
| 70 |  |  |  |  |  |  |  |  |  |  |  |  |  |  |  |  |  |  |  |  |  |  |  |  |  |  |  |  |  |  |  |  |  |  |  | **22** |  |  |
| 71 |  |  |  |  |  |  |  |  |  |  |  |  |  |  |  |  |  |  |  |  |  |  |  |  |  |  |  |  |  |  |  |  |  |  |  | **22** |  |  |
| 72 |  |  |  |  |  |  |  |  |  |  |  |  |  |  |  |  |  |  |  |  |  |  |  |  |  |  |  |  |  |  |  |  |  |  |  | **22** |  |  |
| 73 |  |  |  |  |  |  |  |  |  |  |  |  |  |  |  |  |  |  |  |  |  |  |  |  |  |  |  |  |  |  |  |  |  |  |  | **23** |  |  |
| 76 |  |  |  |  |  |  |  |  |  |  |  |  |  |  |  |  |  |  |  |  |  |  |  |  |  |  |  |  |  |  |  |  |  |  |  | **22** |  |  |
| 79 |  |  |  |  |  |  |  |  |  |  |  |  |  |  |  |  |  |  |  |  |  |  |  |  |  |  |  |  |  |  |  |  |  |  |  | **21** |  |  |
| 82 |  |  |  |  |  |  |  |  |  |  |  |  |  |  |  |  |  |  |  |  |  |  |  |  |  |  |  |  |  |  |  |  |  |  |  | **22** |  |  |
| 85 |  |  |  |  |  |  |  |  |  |  |  |  |  |  |  |  |  |  |  | 19 |  |  |  |  |  |  |  |  |  |  |  |  |  |  |  | **21** |  |  |
| 87 |  |  |  |  |  |  |  |  |  |  |  |  |  |  |  |  |  |  |  |  |  |  | 40 |  |  |  |  |  |  |  |  |  |  |  |  |  |  |  |
| 108 |  |  |  |  |  |  |  |  |  |  |  |  |  |  |  |  | **45** |  |  |  |  |  |  |  |  |  |  |  |  |  |  |  |  |  |  |  |  |  |
| 111 |  |  |  |  |  |  |  |  |  |  |  |  |  |  |  |  | **44** |  |  |  |  |  |  |  |  |  |  |  |  |  |  |  |  |  |  |  |  |  |
| 114 |  |  |  |  |  |  |  |  |  |  |  |  |  |  |  |  | **45** |  |  |  |  |  |  |  |  |  |  |  |  |  |  |  |  |  |  |  |  |  |
| 118 |  |  |  |  |  |  |  |  |  |  |  |  |  |  |  |  | **45** |  |  |  |  |  |  |  |  |  |  |  |  |  |  |  |  |  |  |  |  |  |
| 149 |  |  |  |  |  |  |  |  |  |  |  |  |  |  |  |  |  |  |  |  |  |  |  |  |  | 35 |  |  |  |  |  |  |  |  |  |  |  |  |
| 167 |  |  |  |  |  |  |  |  |  |  |  |  |  |  |  |  |  |  | 38 |  |  |  |  |  |  |  |  |  |  |  |  |  |  |  |  |  |  |  |
| 170 |  |  |  |  |  |  |  |  |  |  |  |  |  |  |  |  |  |  |  |  |  |  |  |  | 29 |  |  |  |  |  |  |  |  |  |  |  |  |  |
| 189 |  |  |  |  |  |  |  |  |  |  |  |  |  |  |  |  |  |  |  |  |  |  |  |  |  | 33 |  |  |  |  |  |  |  |  |  |  |  |  |
| 191 |  |  |  |  |  |  |  |  |  |  |  |  |  |  |  |  |  |  | 34 |  |  |  |  |  |  |  |  |  |  |  |  |  |  |  |  |  |  |  |

Retrospectively analyzed R116G carriers *n* = 14, R116G carriers treated with tofersen in the German Early Access Program (EAP) *n* = 4. Retrospectively analyzed D91A carriers *n* = 8 (*n* = 4 patients with homozygous and *n* = 4 patients with heterozygous allele genotype), D91A carriers treated with tofersen in the German EAP *n* = 3 (*n* = 2 patients with homozygous and *n* = 1 patient with heterozygous allele genotype). Heterozygous allele genotype of D91A is shown in italic. Retrospectively analyzed L145F carriers *n* = 6, L145F carriers treated with tofersen in the German EAP *n* = 3. Patients treated with tofersen in the German EAP are highlighted with bold symbols and ALSFRS-R from the baseline visit on in bold numerals. ALSFRS-R: Amyotrophic lateral sclerosis functional rating scale revised.
